# Vital Measurements of Hospitalized COVID-19 Patients as a Predictor of Long COVID: An EHR-based Cohort Study from the RECOVER Program in N3C

**Published:** 2022-11-15

**Authors:** Sihang Jiang, Johanna Loomba, Suchetha Sharma, Donald Brown, RECOVER consortium, N3C consortium

**Affiliations:** * Department of Engineering of Systems and Environment, University of Virginia; † integrated Translational Health Research Institute of Virginia (iTHRIV), University of Virginia; ‡ School of Data Science, University of Virginia

**Keywords:** Long COVID, vital measurements, time series, summary statistics, machine learning, classification

## Abstract

It is shown that various symptoms could remain in the stage of post-acute sequelae of SARS-CoV-2 infection (PASC), otherwise known as Long COVID. A number of COVID patients suffer from heterogeneous symptoms, which severely impact recovery from the pandemic. While scientists are trying to give an unambiguous definition of Long COVID, efforts in prediction of Long COVID could play an important role in understanding the characteristic of this new disease. Vital measurements (e.g. oxygen saturation, heart rate, blood pressure) could reflect body’s most basic functions and are measured regularly during hospitalization, so among patients diagnosed COVID positive and hospitalized, we analyze the vital measurements of first 7 days since the hospitalization start date to study the pattern of the vital measurements and predict Long COVID with the information from vital measurements.

## I. Introduction

Since the outbreak of COVID-19^1^ pandemic in March 2020, numerous studies focus on the typical symptoms of COVID-19 patients and the characteristic of the transmission process. It is believed that typical symptoms of COVID-19 include fever, dry cough, and fatigue, often with pulmonary involvement, and the incubation period has an average of 5–7 days [1]. The term ‘Long COVID’ is being used to describe the illness in people who have either recovered from COVID-19 but are still reporting lasting effects of the infection or have had the usual symptoms for far longer than would be expected [2]. A research team from Italy studied 143 patients discharged from a Rome hospital after recovering from COVID-19, and found out that 87% were experiencing at least one symptom after 60 days [3]. Common symptoms of Long COVID include profound fatigue, cough, breathlessness, muscle and body aches, chest heaviness or pressure and so on [4], while some patients reported difficulty doing daily activities, in addition to mental health issues [5].

The main risk factors for severe COVID-19 and hospital admission include older age, male sex, non-white ethnicity, disability, and pre-existing comorbidities [6]. However, the risk factors of Long COVID are still generally unclear. Some risk factors of COVID-19 do not increase risk of Long COVID, such as male sex, obesity, diabetes, and cardiovascular disease; pre-existence of asthma has been found to be significantly associated with Long COVID [7]. Scientists have been trying to predict Long COVID with some of the risk factors. According to a recent study, for patients with a duration of COVID symptoms longer than 28 days, five symptoms during the first week that were most predictive were fatigue, headache, dyspnea, hoarse voice and myalgia [7]. Nevertheless, there is limit attention to the vital measurements of hospitalized COVID patients. Compared to symptoms, vital measurements are more frequently measured and available, making wonderful time series to reflect conditions of patients. What’s more, oxygen saturation, heart rate and blood pressure are all quantitative variables, making it easy to be inputs of statistical machine learning models. Thus, exploring the pattern of vital measurements among hospitalized COVID patients in an early stage could help medical workers identify COVID patients with a high risk of Long COVID, give a better understanding of the new disease Long COVID, and make efforts to control the pandemic for social benefits.

## II. Related Work

A recent study analyzed 4,182 incident cases of COVID-19 in which individuals are categorized as short, LC28, LC56 and intermediate [7]. This study applied random forest prediction models using personal characteristics and comorbidities, and the average AUC-ROC was 76.8% in classifying between short COVID and LC28. Some strong predictors include increasing age and the number of symptoms during the first week. The National COVID Cohort Collaborative (N3C) [8] has collected abundant clinical data that can be used to understand the long term effects of COVID-19 and identify the clinical features of Long COVID [9] [10]. In this study, 924 features were selected from demographics, healthcare visits, medical conditions, and prescriptions. An XGBoost model was trained and tested on a set of 97,995 patients who had visited a long COVID clinic, and got an AUC-ROC of 92%. Some of the important features include post-COVID outpatient utilisation, age, post-COVID inpatient utilisation, COVID vaccine and dyspnea [10]. Besides, the social determinants of health (SDOH) [11] are also severely impacting the susceptibility to COVID and Long COVID. Another study has focused on the risk factors associated with PASC [12], including common comorbidities and SDOH factors. As a result, middle age, several specific comorbidities and county level number of doctors are associated with Long COVID. However, there is limited attention to the vital measurements (e.g. oxygen saturation, heart rate and blood pressure) of Long COVID patients, and the pattern of the vital measurements of hospitalized patients are generally not understood. In this paper we study the vital measurements of the first 7 days since the hospitalization start date among COVID and Long COVID patients, and try to use information from vital measurements to predict Long COVID.

Clinical prediction tasks including patient mortality and disease prediction are with much significance for early disease prevention and intervention. A recent study has found that the summary statistics of physiological time series (e.g. min, max, range, mean, standard deviation, skewness, kurtosis)[13] could play an important role in the prediction of length of hospital stay and patient mortality. Blood pressure is an important measure in clinical practice [14], and the variability of blood pressure could imply health conditions. Summary statistics such as standard deviation, skewness and kurtosis could describe the variability of a variable, and thus it is of much interest to study the summary statistics and the time series of vital measurements. As for time series analysis of COVID, various studies have considered the number of cases as time series for forecasting [15], but few studies have taken into consideration the patient level time series, such as the vital measurements.

## III. Methodology

### A. Principal component analysis

Principal component analysis (PCA) is a linear dimension reduction technique in the mean-square error sense [16]. Consider *n* observations of a *p*-dimensional random variable. We denote the observation matrix by ***X***_*p*×*n*_. A linear dimension reduction technique seeks *k* ≤ *p* components of the new variable, being a linear combination of the original variables.

Specifically for PCA, the new variables after the linear transformation are a few variables (the principal components) orthogonal to each other, and linear combinations of the original variables with largest variance. The first principal component has the largest variance, the second principal component is with the second largest variance and orthogonal to the first principal component, and so is this for all other principal components. Ideally, the first several principal components explain most of the variance. As a result of PCA, we transform a *p* × *n* data matrix into a *k* × *n* data matrix, and keep most of the information of the data set in a much lower dimension. It is helpful to understand the data set if different groups have obvious boundaries in a PCA plot.

### B. Kolmogorov–Smirnov test

In statistics, the Kolmogorov–Smirnov test (KS test) is a non-parametric test to compare a sample with a reference distribution (one-sample KS test) [17], or to compare two samples (two-sample KS test) [18]. The one-sample KS test focuses on the probability that the sample is drawn from the reference distribution, and the two-sample KS test focuses on the probability that the two samples are drawn from the same but unknown distribution. The KS test provides a practical tool to compare a sample to a known distribution, or compare samples with each other with unknown distribution.

### C. Machine learning classification

The goal of supervised learning is to approximate a function *f* : *X* → *Y* using a training set $S={\left\{x_{i},y_{i}\right\}}_{i=1}^{N}$ which could describe the relationship between *x* and *y*. Specifically when *Y* = {0, 1}, the this is a binary classification problem. In following we only consider supervised learning, which means the labels are all available [19]. The overall aim of a machine learning problem is to minimize the empirical risk: $\hat{f}=\text{arg}\;{\text{min}}_{f\in F}\frac{1}{N}\sum_{i=1}^{N}L(y_{i},f(x_{i}))$. Commonly used classification techniques in supervised learning include logic-based algorithms (e.g. decision trees), perceptron-based techniques (e.g. neural networks), statistical learning algorithms (e.g. Naive Bayes and Bayesian networks), instance-based learning (e.g. *k*-nearest neighbor) and support vector machines [20]. In this work, we use XGBoost, a scalable machine learning system for tree boosting [21]. This novel tree learning algorithm is suitable for handling sparse data with faster learning process.

### D. Time series methods

#### 1) ARIMA models:

An autoregressive integrated moving average (ARIMA) model is a generalized version of autoregressive moving average (ARMA) model [22]. It is fitted to time series data to better understand the data or to predict future points in the series.

#### 2) Deep learning in time series classification:

Deep neural networks are widely used in time series classification tasks, such as Multi Layer Perceptron (MLP), Convolutional Neural Network (CNN), Echo State Network (ESN) [23] and long short term memory Recurrent Neural Network (LSTM RNN) [24].

## IV. Results and analysis

As of completion of the paper, the N3C cohort [25] contains 5,274,332 patients with an active COVID-19 infection as indicated by a U07.1 code or a positive PCR or AG SARS-CoV-2 lab test, the first instance of which we use as their COVID-19 index date. Of these, 327,964 patients were hospitalized in the day prior to 16 days following the index SARS-CoV-2 PCR or AG lab result and a COVID-19 diagnosis of U07.1 was recorded in that same time period. Efforts have been made to harmonize the units and values from electronic health records in N3C [26], and in the following section we study vital measurements as shown in Table I.

To study how the emergence of Long COVID may be predicted by pattern of vital measurements of the 327,964 hospitalized patients we needed to identify a Long COVID indicator. Due to limited documentation of Long COVID, we follow the work in [27], where the Long COVID indicator is derived from a machine learning-based computable phenotype definition trained on cases where the U09.9 (Long COVID) diagnosis code was recorded. The computable phenotype as-signs a likelihood score between 0 and 1, and in the following analysis, patients with computable phenotype values larger or equal to 0.75 are labeled as ‘Long COVID’, and patients with computable phenotype values smaller or equal to 0.25 are labeled as ‘non Long COVID’. Because only a subset of patients are assigned values in these two ranges, we end up with a cohort of 85,196 patients who are all hospitalized around the time of the first known COVID infection and who we can assign a binary value for our Long COVID indicator.

### A. Summary of the cohort

In this cohort of 85,196 patients, the average and the median of the age at the time of first known COVID-19 infection are57.5 and 60, white non-Hispanic patients consist of 55.4% of the cohort, female patients consist of 51.75% of the cohort, and 33.52% patients of the cohort are Long COVID patients.

### B. Vital measurements of the cohort of hospitalized patients

To study the pattern of the vital measurements of Long COVID patients, we extract the vital measurements readings from the relevant COVID-associated hospitalizations. The majority of the cohort have a short length of hospitalization, with an average of 8.1 days, and a median of 4 days. In order to reduce the impact of variable length of stay, we chose to focus on vitals collected during the first week of hospitalization. Before creating features from the vital measurements readings, we try to explore the distribution and the richness of the vital measurements readings.

**Fig. 2:**
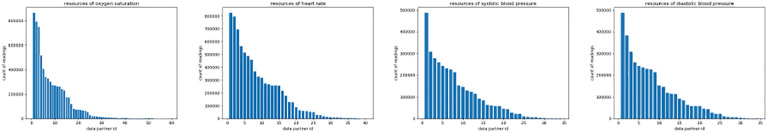
Resources of readings of the cohort

**Fig. 3:**
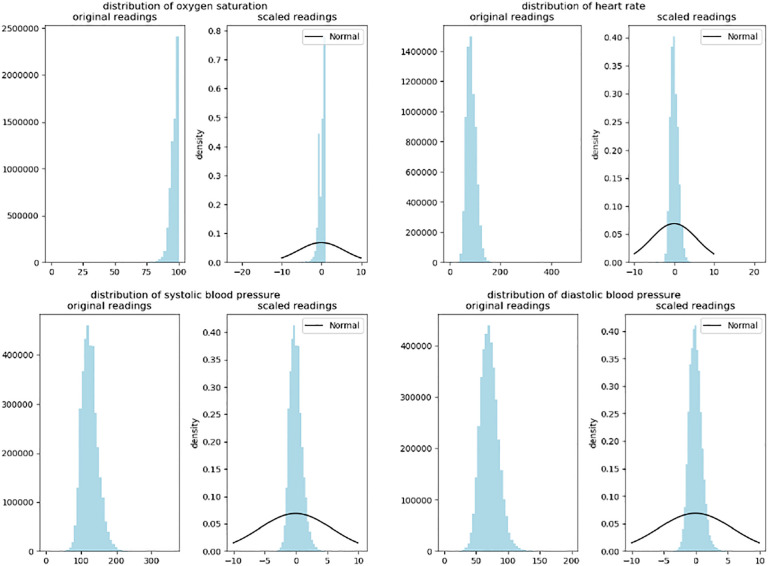
Distribution of readings of the cohort

In the N3C cohort, records of patients are provided by anonymized institutions, represented by a variable ‘data partner id’. To comply with N3C policy, the data partner ids in Fig. 2 don’t represent the real resources, and same data partner id belonging to different vital measurements might represent different resources. Table II shows the amount of available readings of each vital measurement, and the number of patients that these readings belong to. Fig. 3 shows that the distribution of each vital measurement has a very high peak, and the distribution of oxygen saturation readings is less symmetric than the other 3 vital measurements. A KS test is performed on each vital measurement to test whether the readings are from a normal distribution, and the *p*-values of the KS test on each vital measurement are all 0, so we reject the null hypothesis that the readings are from a normal distribution.

With vital measurements readings in the first 7 days since the hospitalization start date, features describing overall conditions of the vital measurements are created as following:

1. **Daily averages**: Respectively for each vital measurement, we take the daily average of readings of each patient for 7 days, and get a series with length 7.
2. **Overall summary statistics features**: Respectively for each vital measurement, we calculate the summary statistics (e.g. min, max, median, quartiles, range, standard deviation, skewness and kurtosis) of all readings of each patient.
3. **Daily variability features**: Respectively for each vital measurement, we first calculate the daily min, daily average, and daily max, and then calculate the variability measure (standard deviation, skewness, kurtosis) of the daily min, daily average and daily max of each patient.

### C. Subcohort with rich data

Despite the widely used electronic health record data, there are still only a relatively small portion of patients among the hospitalized patients with available vital measurements data, as Table II shows. Thus, we select subcohorts from hospitalized patients with rich data for further numerical analysis of the vital measurements, especially the prediction of Long COVID using features created from vital measurements.

#### 1) Subcohort A:

The subcohort A consists of 16,468 patients with at least one reading of each of the four vital measurements (oxygen saturation, heart rate, systolic blood pressure, diastolic blood pressure). There are 8,385 female patients in the subcohort A, and among all patients in the subcohort A, 6,088 patients are labeled as ‘Long COVID’, and the average and median of age of subcohort A are 56.5 and 59. Table III shows the overall richness of vital measurements readings in 7 days of subcohort A.

**Fig. 4:**
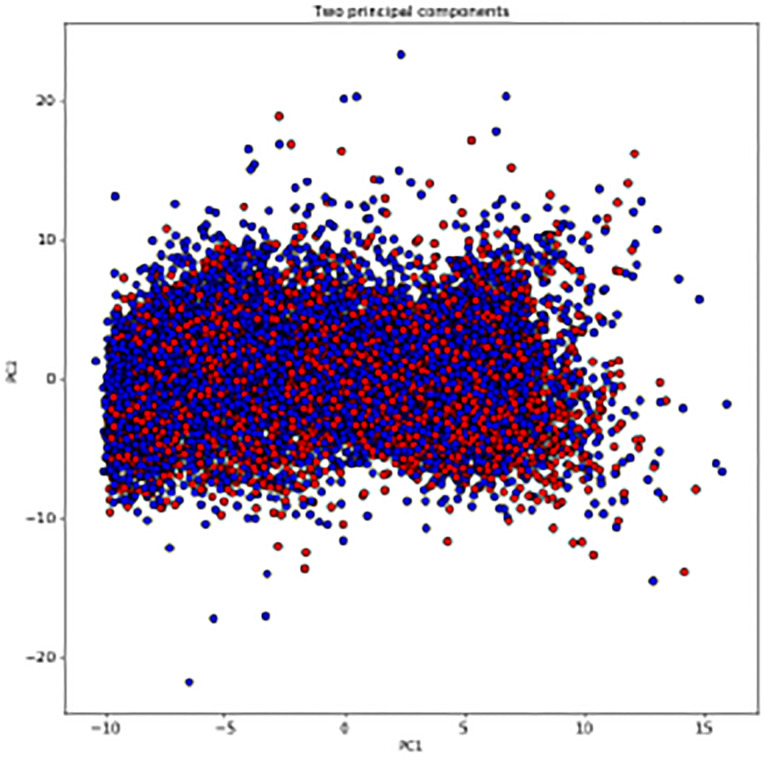
PCA analysis of the subcohort A

To get more information from the vital measurements, we focus on the feature set of vital measurements including daily averages, overall summary statistics and daily variability features of the four vital measurements (oxygen saturation, heart rate, systolic blood pressure, diastolic blood pressure). This feature set of vital measurements of subcohort A has 139 features, and a principal component analysis is performed on the vital measurements feature set. As Fig. 4 shows, red dots represent Long COVID patients, and blue dots represent non Long COVID patients in subcohort A, and the two principal components explain 19.74% and 8.90% of the total variance of the original feature set of vital measurements. It is desirable that there is an obvious boundary between these two groups.

Besides, a KS test is performed on the feature set of vital measurements as well. The subcohort A is divided to two groups: the group of Long COVID patients and the group of non Long COVID patients. The KS test is to test whether the features under these two groups are from the same distribution. A feature with a large *p*-values means that we fail to reject the null hypothesis that the samples of this feature in two groups are from the same distribution. As a result, there are 118 features with *p*-value smaller than or equal to 0.01, and Table IV shows 4 features with a *p*-value larger than 0.1.

As a result of the PCA and KS test, the two principal components could not separate the group of Long COVID and the group of non Long COVID, and most features have different distributions in these two groups. To better understand the role of vital measurements in the prediction of Long COVID, we train XGBoost models on the subcohort A respectively using 3 feature sets, where feature set 1 is referring to the risk factor analysis of Long COVID, but without age, gender and race and ethnicity of the cohort so as to avoid overlap with the feature set used to generate our Long COVID labels [12]:

- **Feature set 1 (SDOH and pre-COVID conditions)**: 51 features including social determinants of health (SDOH) and pre-COVID health conditions
- **Feature set 2 (vital measurements):** 139 features created from vital measurements as mentioned above, including daily averages, overall summary statistics and daily variability features
- **Feature set 3 (SDOH, pre-COVID conditions and vital measurements):** 190 features combining feature set 1 and feature set 2

With 5-fold cross validation, we evaluate performance of the models with the common metric, area under the ROC curve (AUC) and the F1 score (the harmonic mean of precision and recall) [28]. Also, we calculate the permutation importance of each feature. As Table V shows, in the subcohort A with rich vital measurements data, the vital measurement features perform better than the SDOH and pre-COVID conditions in the prediction of Long COVID, and adding the vital measurements features to SDOH and pre-COVID conditions could further improve the performance of the XGBoost model in prediction of Long COVID. In addition, we show the top 10 important features of each XGBoost model:

- **Feature set 1 (SDOH and pre-COVID conditions):** extended stay, chronic lung disease before COVID, Corticosteroid before COVID, dementia before COVID, hypertension before COVID, obesity before COVID, depression before COVID, percent insured 65 plus public, Corticosteroid during COVID hospitalization, long stay
- **Feature set 2 (vital measurements):** measurement duration of systolic blood pressure, measurement duration of diastolic blood pressure, observation per hour of diastolic blood pressure, measurement duration of oxygen saturation, minimum measurement time of heart rate since hospitalization, observation per hour of oxygen saturation, average of oxygen saturation, measurement duration of heart rate, maximum measurement time of heart rate since hospitalization, average of heart rate on the third day
- **Feature set 3 (SDOH, pre-COVID conditions and vital measurements):** measurement duration of systolic blood pressure, chronic lung disease before COVID, extended stay, dementia before COVID, Corticosteroid before COVID, depression before COVID, Corticosteroid during COVID hospitalization, average of heart rate on the first day, measurement duration of diastolic blood pressure, measurement duration of oxygen saturation

#### 2) Subcohort B:

The subcohort B consists of 5,304 patients with available daily averages of each of the four vital measurements (oxygen saturation, heart rate, systolic blood pressure, diastolic blood pressure) for first 7 consecutive days since the hospitalization start date. In other words, patients in subcohort B have 4 dimensional time series with length7. There are 2,392 female patients in the subcohort B, and among all patients in the subcohort B, 2,237 patients are labeled as ‘Long COVID’, and the average and median of age of subcohort B are 63.3 and 65.

**Fig. 5:**
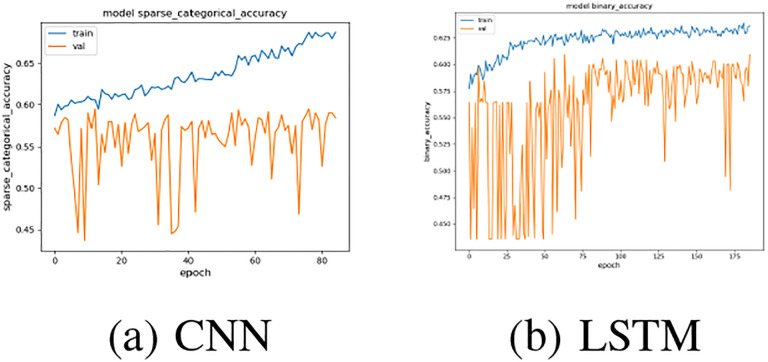
Results of CNN and LSTM models

To get an understanding of the trend of series, a regression model between average oxygen saturation value of 7th day and values of first 6 days is fitted on subcohort B. This regression model has a mean sqaured error of 2.615 and a *R*^2^ score of 0.5691. Because of the low *R*^2^ value, this regression model seems not relatively informative with a weak linear relationship, and the relationship inside the time series is still underneath the hood. Using the daily averages as features, common machine learning classification models are trained and evaluated as Table VI shows. Two neural network models are trained on subcohort B: one is using a fully convolutional neural network [29] with 3 convolutional layers followed by the global average pooling process, and the other is using a layer of LSTM [30]. As a result, this CNN model has a test accuracy 0.6164 and a test loss 0.6673, and the LSTM model has a test accuracy of 0.5994 and a test loss of 0.6707. Fig. 5 shows the training loss and the validation loss with the number of epochs.

### D. Conclusion

In the analysis of the cohort of Long COVID, the relationship between vital measurements and Long COVID has caught much attention. This cohort is balanced in age, gender and race and ethnicity, but not all of patients in this cohort have available data of vital measurements. Thus, two subcohorts with rich vital measurements data are created. Because the average and median of hospitalization length are short for most patients, we focus on the vital measurements in the first 7 days since the hospitalization start date, and various features are created from the vital measurements readings. The feature set of vital measurements has 139 features, including the daily averages, the summary statistics of the vital measurements and the daily variability features. PCA is a great method to reduce the dimension of the data set, as well as the size of the feature set. Using the SDOH and pre-COVID conditions, vital measurements and a combination of these two as feature sets, the XGBoost model is trained on the subcohort A and gives a prediction of Long COVID. As a result, the feature set of vital measurements outperforms the feature set of SDOH and pre-COVID conditions, and the combined feature set could further improve the accuracy of the prediction. The subcohort B contains time series of the vital measurements, making possible the use of neural networks in time series classification. CNN and LSTM are great tools of processing multidimensional time series. In summary, in subcohorts with rich vital measurements data, the vital measurements are informative and with significance in the analysis of Long COVID.

## V. Limitations and future work

This paper gives a summary of explorations on the vital measurements of patients in the hospitalized Long COVID cohort, including the extracted features from vital measurements and the prediction of Long COVID using these features. However, since the Long COVID is still a new disease, there is only a small portion of patients with available vital measurements data in the whole N3C cohort, and more comprehensive data is desired. Besides, the biomedical meaning of the features created from vital measurements readings, such as the summary statistics of the time series, are generally unclear, so it is important to understand which features are medically significant. With an overall goal of predicting Long COVID with features created from vital measurements, we always need appropriate classification techniques that could predict Long COVID more accurately, including machine learning methods and deep neural networks. Methods of selecting models should be included in further analysis. While training models, the choice of hyperparameters would significantly influence the performance of models, and common methods of selecting hyperparameters include random search and grid search [31]. These methods should be taken into consideration for better results.

## Figures and Tables

**Fig. 1:**
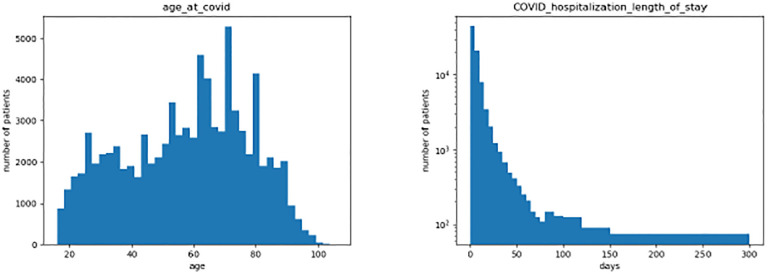
Age summary and length in hospital of the cohort

**TABLE I:** Vital measurements

Measured variable	Unit	Range
SpO2 (oxygen saturation)	Percent	0~100
Heart rate	Bpm	0~500
Systolic blood pressure	mmHg	0~400
Diastolic blood pressure	mmHg	0~200

**TABLE II:** Availability of vital measurements of the cohort

Measurements	Readings	Patients	Resources
Oxygen saturation	6,959,178	44,479	59
Heart rate	7,833,270	44,253	40
Systolic blood pressure	3,530,787	32,706	34
Diastolic blood pressure	3,840,918	35,628	34

**TABLE III:** Number of readings per patient of subcohort A

Measurements	Average number of readings per patient	Median number of readings per patient
Oxygen saturation	49.4	23
Heart rate	53.2	24
Systolic blood pressure	39.3	23
Diastolic blood pressure	38.8	23

**TABLE IV:** KS test on subcohort A

Feature	*p*-value
third_quartile_dia_bp_7_day	0.1057
mean_dia_bp_1	0.2530
mean_dia_bp_2	0.2058
mean_sys_bp_1	0.1621

**TABLE V:** XGBoost Results

Feature set	Mean AUC (5-fold CV)	Mean F1 score (5-fold CV)
Feature set 1	0.729 ± 0.008	0.696 ± 0.007
Feature set 2	0.787 ± 0.007	0.717 ± 0.007
Feature set 3	**0.822 ± 0.007**	**0.752 ± 0.005**

**TABLE VI:** Machine learning models on subcohort B

Method	Mean AUC (5-fold CV)
XGBoost	0.614 ± 0.015
Logistic regression	0.614 ± 0.015
Support vector machine	0.617 ± 0.016
Naive Bayes	0.608 ± 0.017
k-nearest neighbor (k=5)	0.550 ± 0.014

## Footnotes

1 The RECOVER’s official terminology of COVID-19 is SARS-CoV-2.
